# Characterization of Microparticle Separation Utilizing Electrokinesis within an Electrodeless Dielectrophoresis Chip

**DOI:** 10.3390/s130302763

**Published:** 2013-02-27

**Authors:** Chi-Han Chiou, Jia-Cheng Pan, Liang-Ju Chien, Yu-Ying Lin, Jr-Lung Lin

**Affiliations:** 1 ITRI South Campus, Industrial Technology Research Institute, Tainan 70955, Taiwan; E-Mails: prayjohn@itri.org.tw (C.-H.C.); liangju@itri.org.tw (L.-J.C.); yuyinglin@itri.org.tw (Y.-Y.L.); 2 Department of Mechanical and Automation Engineering, I-Shou University, Kaohsiung 84001, Taiwan; E-Mail: jiacheng.pan@innolux.com

**Keywords:** separation, electrodeless dielectrophoresis, electroosmosis, dielectrophoresis

## Abstract

This study demonstrated the feasibility of utilizing electrokinesis in an electrodeless dielectrophoresis chip to separate and concentrate microparticles such as biosamples. Numerical simulations and experimental observations were facilitated to investigate the phenomena of electrokinetics, *i.e.*, electroosmosis, dielectrophoresis, and electrothermosis. Moreover, the proposed operating mode can be used to simultaneously convey microparticles through a microfluidic device by using electroosmotic flow, eliminating the need for an additional micropump. These results not only revealed that the directions of fluids could be controlled with a forward/backward electroosmotic flow but also categorized the optimum separating parameters for various microparticle sizes (0.5, 1.0 and 2.0 μm). Separation of microparticles can be achieved by tuning driving frequencies at a specific electric potential (90 Vpp·cm^−1^). Certainly, the device can be designed as a single automated device that carries out multiple functions such as transportation, separation, and detection for the realization of the envisioned Lab-on-a-Chip idea.

## 1. Introduction

In recent decades, cell or microparticle separation has attracted significant attention in sample preparation for biological and chemical applications, especially in microfluidic systems. Several particle manipulation approaches have been employed, including dielectrical [1], magnetic [2], and optical [3] manipulations. Each of these methods has its advantages and disadvantages. For example, electrode-embedded array dielectrophoresis (DEP) [4,5] provides many advantages such as flexibility, controllability and ease of application, and it has been proven to be an efficient non-invasive method for separating various cell types without any need for labeling. However, the inherent characteristics of the electrode-embedded array DEP not only lead to fast decays of field gradient but also hardly allow the ability of electroosmotic (EO) transport for conveying particles unless more complicated microdevice designs are involved [6], thus significantly reducing the trapping efficiency and the microparticle separation throughput. Magnetic manipulation can be used to separate microparticles; however, particles with slight size variations and magnetization are difficult to separate. Meanwhile, applying a higher magnetic force could result in a higher device temperature, which in turn could destroy biosamples. Although optical manipulation is non-contact and contamination-free, the complicated optical setup, complex operation, and expensive instrumentation further limit its application in microfluidics.

Currently, one concept of the DEP method is insulator-based (electrodeless) DEP (iDEP or EDEP), in which a high field gradient is generated at an insulating constriction in a conducting solution, avoiding problems in some of the electrode-embedded array DEP [7,8]. EDEP can be performed using different geometries and configurations of insulating structures, such as triangular constricted structures [8–10], circular posts [7,11,12], rectangular barriers [13,14], nanopipettes [15,16], and oil drops [17]. Among these configurations, the triangular constricted structure is more attractive because the field gradient can be preserved over the entire cross section of the trap [8–10]. This feature is crucial for efficient particle trapping and quantitative determination of the concentrated particles for trace analysis. Although an increasing number of studies exploit EDEP, it does not allow effective manipulation of particles in a microfluidic channel without a full understanding of their electrokinetic behaviors. Numerous EDEP parameters including the geometry of the insulating structure, material properties, applied electric field and driving frequency must be evaluated. These factors simultaneously affect the physics phenomena of electrokinesis (EK).

Electrokinesis phenomena commonly occur in microfluidic applications, in which particles and liquids are separated, transported, and especially concentrated. Concentration is a critical sample-processing step for clinical and environmental sample analysis [9,18,19]. Basically, EK involves two important microfluidic mechanisms. One is called dielectrophoresis, which is the migration of particles with an induced dipole moment in a non-uniform electric field caused by an electric field gradient. The DEP force has been exploited for trapping, fusing, sorting, and lysing biological samples [9,12,20]. The other is electroosmosis flow (EOF), which is a fluid flow that is caused by a net momentum transfer of charges to the fluid within an electric double layer (EDL). The EOF drag force is typically utilized to convey both particles and liquids [7,21]. Recently, biomolecules or particles were continuously trapped and collected in a world-nano-micro interface using an EOF transport mechanism [22,23]. Both EOF and DEP are established by applying an electrostatic field to a conductive fluid, and they coexist in a microchannel. The magnitudes of the EOF and DEP effects on particles and liquids depend on numerous physical and chemical properties of the solid surface, the particles, the medium, and even the geometry of the microchannel [7,11,24]. Based on these properties, various derivative applications of particle manipulations and liquid conveyance using DEP or EOF have been developed. In EDEP applications, the transport of a fluid generated by EOF is eliminated or minimized by increasing the fluid viscosity or modifying the surface properties of the channel [8]. However, EDEP applications have seldom been adopted to analyze how DEP can concentrate particles when EOF occurs in a microchannel. Although EDEP is ideal for trapping particles, the region affected by DEP is restricted to the area of high field gradient close to the constricting gap, resulting in lower efficiency of sample concentration.

In this study, an EDEP microfluidic chip is demonstrated to successfully separate three groups of microparticles. Microparticles of different sizes can be easily separated at different locations in the EDEP microfluidic chip. The investigation of EK behaviors with numerical simulations and experimental observations reveals that microparticle separation is significantly dominated by driving frequencies. Tuning driving frequency allows the separation of microparticles of various sizes in consecutive order without an external transport system.

## 2. Theory

### 2.1. Dielectrophoresis

For spherically polarized particles transported in a conductive medium under a non-uniform electric field, the DEP force exerted on the particles is expressed by the following equation [25]:

(1) FDEP=2πa3ɛmRe[CM]∇E2

where *a* is the radius of the particle; ε_m_ is the permittivity of the medium; and *E* is the amplitude of the electric field. Re[*CM*] is the real part of the complex Clausius-Mossotti factor. Basically, the DEP force, *F_DEP_*, is proportional to the gradient of the square of the applied electric field and to the third power of the particle radius. Here, the complex Clausius-Mossotti factor (*CM*) was obtained by:

(2) $$CM=\frac{{ɛ}_{p}^{*}-{ɛ}_{m}^{*}}{{ɛ}_{p}^{*}+2{ɛ}_{m}^{*}}$$

(3) $${ɛ}^{*}=ɛ-j\frac{\sigma }{w}$$

where, ε* is the complex permittivity; σ is the conductivity; *w* is the angular frequency of the electric field; and *j* is the imaginary unit. The subscripts *p* and *m* refer to the particle and the medium, respectively. In principle, the real part of Re[*CM*] is bounded between 1.0 and −0.5. In this study, the *Re*[*CM*] factor was calculated to be approximately −0.49, as the frequency ranged from 10 Hz to 10 MHz.

### 2.2. Electroosmosis

An external tangential potential was applied to the conductive medium to establish localized zeta potential variations within the electrical double layer (EDL). Accordingly, excess ions inside the diffuse double layer experience a Stokes drag force that, on average over time, acts on the fluids to move them forward or backward. Assuming that spherical particles travel into the viscous medium, the Stokes drag force (*F_st_*) is given by the following equation:

(4) $$F_{st}=6\pi \eta a(u_{\text{EOF}}-u_{p})$$

where *u_EOF_* and *u_p_* denote the electroosmotic flow velocities of the medium and the microparticle, respectively. The Stokes force is linearly proportional to the velocity and the particle radius. The electroosmotic flow velocity (u_EOF_) can be represented as:

(5) $$u_{\text{EOF}}=\frac{-{ɛ}_{m}\zeta E_{x}}{\eta }$$

where η is the medium viscosity; ζ is the zeta potential of the microchannel wall; *E_x_* is the x-directional electric field strength and is also strongly a function of the applied voltage as well as the driving frequency. Zeta potential (ζ) can be expressed as shown below [26]:

(6) $$\zeta =\frac{2k_{b}T}{ez}\sinh^{-1}\left(\frac{\lambda_{D}{ɛ}_{s}(E_{y}-E_{yo})}{4n^{o}ez}\right)$$

Here, *k_b_* is the Boltzmann constant; *T* is the absolute temperature; *e* is the electronic charge; *z* is the ionic valence; *n*° is the ion concentration; λ_D_ is the inverse Debye length; *ε_s_* is the permittivity of the PDMS (polydimethylsiloxane); *E_y_* is the component of the electric field in the y direction; and *E_yo_* is the virtual electric field that yields the initial value of the zeta potential arising from the initial charge density on the PDMS channel surface. The value of *E_yo_* was then calculated to be 1.5 × 10^5^ V/m in this study.

### 2.3. Electrothermosis

A numerical simulation and an experimental evaluation were conducted to investigate the distribution of the temperature field inside the EDEP chip. Because the fluid flow rate of the liquid in the EDEP channel was slightly faster, particularly in the constricted gap, it was reasonable to assume that the heat transfer was dominated by heat conduction and convection. Therefore, in this study, heat radiation was neglected, and heat conduction and convection within the liquid sample, the solid PDMS channel wall, and the glass substrate was included. Here, a 3-D heat conduction and convection model was used to describe the thermal phenomena. The governing equation is given by:

(7) $$\rho C_{P}\left(\frac{\partial T}{\partial t}+\vec{V}\cdot \nabla T\right)=\nabla \cdot \left(k\nabla T\right)+\sigma {\vec{E}}^{2}$$

Where, ρ is the mass density of the fluid; *k* is the thermal conductivity; *Cp* is the specific heat; *V* is the fluidic velocity; and σE^2^ is the Joule heating term.

A three-dimensional (3-D) numerical simulation was then conducted to investigate and characterize the distributions of temperature effects and the DEP force under the non-uniform AC electric fields. The electromagnetic physical properties of an EDEP microfluidic chip were numerically calculated using the commercial computational fluidic dynamics (CFD) software (CFD-ACEU, CFD-RC, Huntsville, AL, USA). A multi-physics coupled analysis (*i.e.*, flow-, thermal-, spray-, and electric-module) were conducted into this simulation. Table 1 lists the physical properties of the materials that were assumed in the numerical simulations.

## 3. Materials and Methods

### 3.1. Design and Fabrication

**Figure 1.**
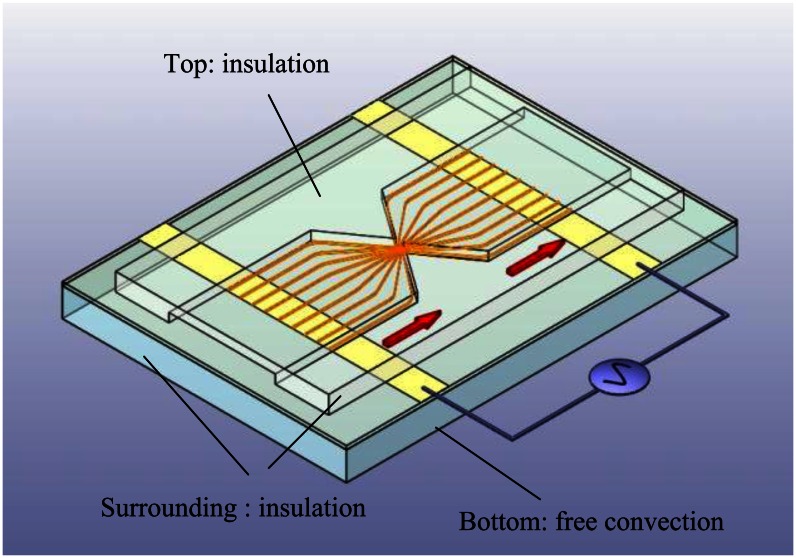
Schematic EDEP-based microfluidic device, comprising a pair of triangular insulating structures, a microchannel, and a pair of driving electrodes. Additionally, boundary conditions of the 3-D numerical simulation described in a triangular insulating structure are shown.

The scheme of the proposed EDEP microfluidic device comprising a pair of triangular insulating structures, a microfluidic channel, and a pair of driving electrodes is shown in Figure 1. Two 60°-triangular insulating structures constructed a constricting gap and squeezed the electric field in a conductive solution to produce a high electric field gradient for trapping particles.

In a highly non-uniform electric field caused by a constricted gap, polarized particles experience a force in the direction along (positive DEP, pDEP) or against (negative DEP, nDEP) the electric field gradient, depending on the dielectric properties of the particles. A pair of driving electrodes separated by 2 mm was adopted to produce the required electric field in the microchannel. The microchannel was 1,000 μm wide and 5 μm deep. The 5 μm constriction gap was operated at a low flow resistance to examine the interdependence between the EO and DEP effects.

**Figure 2.**
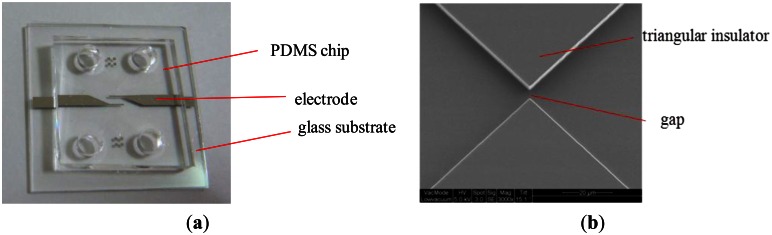
(**a**) A photograph of an assembly of an EDEP-based chip. (**b**) An optical microscope image of the triangular insulating structures with a 5 μm constriction gap.

The EDEP microfluidic chip was fabricated using the Micro-electromechanical Systems (MEMS) technology. Two layers of Ti/Pt (200 Å/2,000 Å) were deposited on a glass substrate to form a pair of electrodes by using the lift-off process. A silicon wafer was patterned using standard photolithography, reactive ion etching (RIE) was employed to produce a 5 μm-deep mold, and the mold was cast with a pair of triangular insulating structures and a microfluidic channel. Accordingly, the inverse structures with patterned features were cast using PDMS materials and the Si mold. Finally, the replicated PDMS structures and the glass substrate with Pt electrodes were bonded to each other with oxygen plasma treatment to yield a complete EDEP microfluidic chip. Figure 2(a) shows the assembly of the EDEP chip. Figure 2(b) displays the scanning electron microscope (SEM) image of the Si mold of the 5 μm constriction gap.

### 3.2. Experiment

The experimental setup involved a signal generator (33220A, Agilent Technologies, Santa Clara, CA USA) and an oscilloscope (TDS 1002B, Tektronix, Beaverton, Oregon, OR, USA). A power amplifier (LPA 400, Newtons4th Ltd, Mountsorrel, Charnwood, UK) was employed for the investigation of the EDEP microfluidic chip. The EDEP microfluidic chip was mounted on top of an inverted microscope (DMI 4000B, Leica, Germany) for microparticle visualization. The polystyrene microparticles (excitation 580 nm/emission 605 nm, Invitrogen, Carlsbad, CA, USA) were used to experimentally investigate the electrokinesis effect. Fluorescence images of the microparticles were captured using a 10× or 20× objective lens and a cool Charge-coupled Device (CCD, Cool SNAP HQ^2^, Photometrics, Huntington Beach, CA, USA). Moreover, the solution viscosity was adjusted to 1 cP with a buffer solution of 10 mM Tris-HCl (pH 8.0) having a fixed conductivity of 730 μS·cm^−1^. In all experiments, the electric field was fixed at 90 V_pp_·cm^−1^, and the driving frequencies were in the range of 10 Hz to 5 MHz.

## 4. Results and Discussion

### 4.1. Numerical Characterization

**Figure 3.**
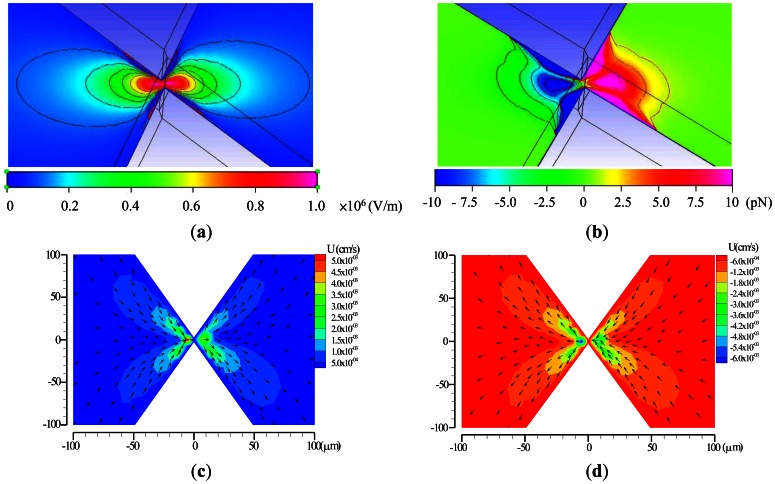
Numerical contours of the (**a**) *E_x_* and (**b**) *DEP* force at the applied field of 90 V_pp_·cm^−1^ with a driving frequency of 1 kHz. Numerical results of (**c**) forward EOF at a driving frequency of 1 kHz and (**d**) backward EOF at a driving frequency of 5 MHz.

To test the functions of the EDEP microfluidic chip, a simple insulating-based cell, defined in a microfluidic channel, as shown in Figure 1(b), was considered. In an electric field of 90 V_pp_·cm^−1^, driving frequencies ranging from 10 Hz to 5 MHz were applied as a sinusoidal wave across the microchannel through a constriction gap. The contours of the x-directional electric field (*E_x_*) were non-uniform as presented in Figure 3(a). The electric field in the ionic solution was squeezed in the constricted region. Hence, the electric field gradients were highly non-uniform around the two constricted regions, especially at the walls of the constriction gap. Figure 3(b) explores the distribution of the DEP force between the triangular insulators. The blue color indicates the n-DEP force, and the red color represents the p-DEP force. Certainly, these values can only be influenced by varying driving frequencies and particle sizes. The numerical results can explain the semicircular shapes of microparticle trapping contours and that particle trapping begins in the constriction wall. The applied field of 90 V_pp_·cm^−1^ and the driving frequency of 1 kHz were used in the numerical simulation. Figure 3(c,d) shows numerical velocity contours at a forward and backward EOF, respectively. The EOF effect occurred between a pair of triangular insulating constructions, and higher velocities occurred in the constriction gap. The simulated results were in agreement with the experimental observations.

**Figure 4.**
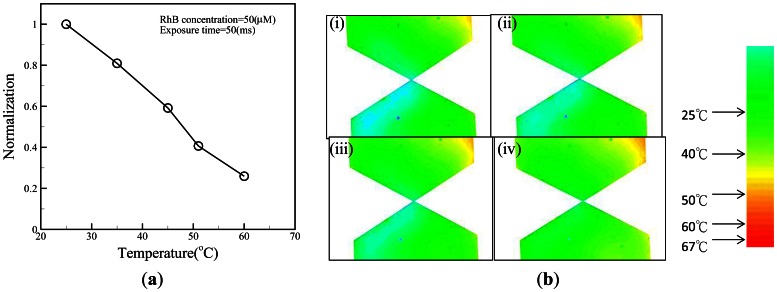
(**a**) Normalized fluorescence intensity is plotted as a function of temperature and is used to calibrate the fluorescence-based temperature measurement. (**b**) False color image of the buffer temperature for electroosmotic flow through a microchannel with an applied voltage V = 90 V_pp_·cm^−1^ and a frequency f = 1 kHz. Temperature distribution images (i-iv) were taken 3, 10, 20, and 30 min after switching on the device.

The temperature effect is another important issue for biosamples, as elevated temperature can damage biosamples. Here, a highly temperature-sensitive fluorescent dye (rhodamine B) was used to determine the fluid temperature in a microchannel [27]. A rhodamine B solution (50 μM) was used to calibrate the temperature measurement by averaging the intensity values (after background subtraction) of all the pixels of the corresponding image (Figure 4(a)). Once the calibration curve had been determined, it was used to measure the temperature of the solution inside a microchannel by comparing the fluorescence intensity at an unknown temperature to the intensity at room temperature (25 °C). To confirm whether the applied voltage might cause increased temperature in the microchannel, a time series of temperature distributions in the microchannel under the fixed voltage was measured (Figure 4(b)). The results showed that the fluid in the triangular insulators could be maintained at 25 °C even 30 minutes after switching the voltage on, as shown in Figure 4(b). However, it was difficult to make experimental observations at the gap of the triangular insulators because the gap region was too small. To investigate the temperature field distribution inside the EDEP chip, a numerical simulation was carried out. A 3-dimensional (3-D) thermal model was used to describe the phenomena. The area of the surrounding surface (*i.e.*, EDEP thickness) was much less than that of the top or bottom surface. Certainly, the heat was mainly dissipated from the top (solid PDMS wall) or bottom (glass). Additionally, the thermal conduction of a solid PDMS cover was also much lower than that of a glass substrate. Therefore, the natural free convection condition was imposed on the bottom glass surface and insulated on the top solid PDMS wall and the surrounding surfaces of the EDEP chip. Notably, the heat convection was taken into account in this simulation. The boundary condition is depicted in Figure 1.

**Figure 5.**
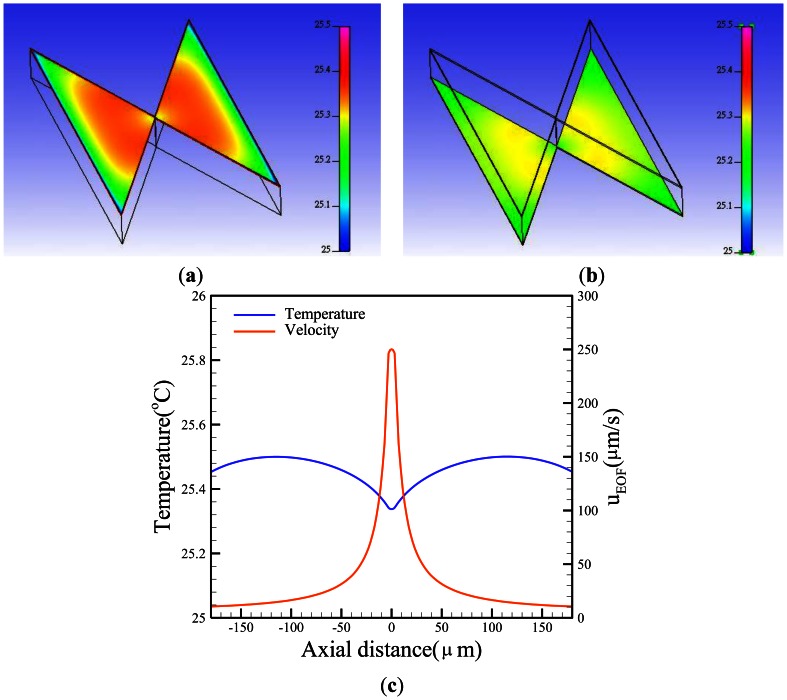
Numerical calculations of the (**a**) top and (**b**) bottom temperature contours of insulators and (**c**) the central x-axial temperature, as well as velocity profiles at the applied potential V=90 V_pp_·cm^−1^ and the frequency f = 1 kHz.

Free convection effects are difficult to determine due to their complexity. The convection heat transfer coefficient (*h*) is a function of the surface geometry, the nature of fluid motion, the properties of the fluids, and the bulk fluid velocity. In this simulation, the value of h was set to 7.5 Wm^−2^·K^−1^[28] to evaluate the convective heat transfer. An assumed heat generation rate was applied to the AC potential (*σE*^2^). Figure 5(a) shows the temperature contours of a paired triangular insulator. Basically, the temperature is proportional to the square of the electric field. Certainly, the higher the electric field, the higher the temperature gets. The temperature distribution demonstrated a slight difference (∼0.4 °C) from the surrounding temperature. Conversely, the temperature of the gap was slightly lower than the other region, resulting in a higher EOF velocity due to the heat convection as shown in Figure 5(c).

### 4.2. EOF Characterization

**Figure 6.**
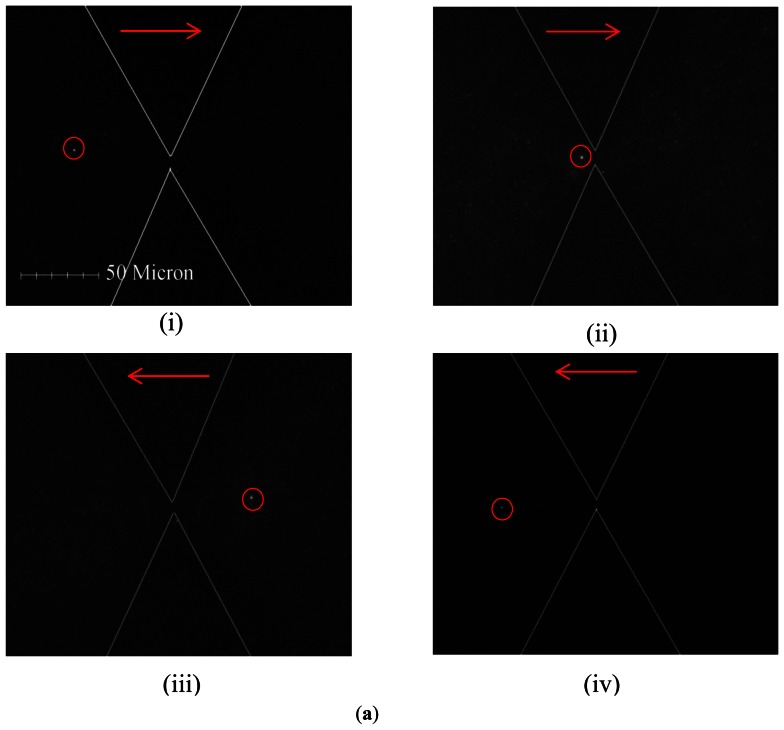

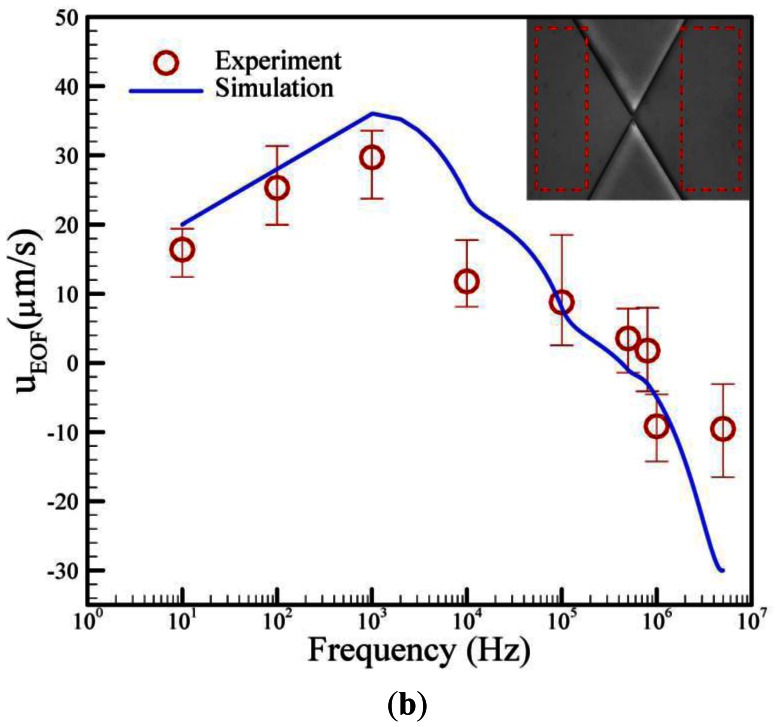
(**a**) Conveyance of 0.2-μm particles under electroosmosis. The red arrow indicated the flow direction, and a 1.0-μm microparticle (marked with a red circle) referred to the forward/backward flow direction. (**b**) The numerical and experimental relations between EOF velocities and different frequencies. Notably, the two red dashed-line regions shown in Figure 6(b) indicate the numerical and experimental mean EOF velocities.

Theoretically, two balanced forces are exerted on the trapped particles. The first force is the DEP force (*F_DEP_*), which acts as a holding force to stop the particles from moving. The other force is Stokes force (*Fst*), which acts on the particles through EO fluid and aids transport. Therefore, the net motion of particles is determined by both the EO and DEP effects. Figure 6(a) shows that the fluorescent 0.2 μm particle passed through the constriction when a field with a frequency from 10 Hz to 5 MHz was continuously driven at the constant voltage of 90 V_pp_·cm^−1^. The microparticles flowed forward through the constriction gap by EO conveyance and were not trapped by the DEP effect when the driving frequency was lower than 810 kHz. Conversely, the flow direction reversed (backward flow) when the driving frequency was higher than 810 kHz. The EOF effect also occurred between a pair of triangular insulating constructions. Higher velocities were also shown near the constriction walls because of the zeta potential effect. Interestingly, a reciprocating motion was observed at a frequency of 810 kHz. Summarily, the numerical and experimental relationship of the EOF velocity and the driving frequencies was plotted as Figure 6(b). It was clearly observed that the EOF velocity initially increases with the operating frequency, and then, EOF velocity reaches a maximum value and subsequently decreases to become negative as the operation frequency is increased further. The numerical results were also revealed to be reasonably in agreement with the experimental measurements. The numerical simulation is a powerful tool for the investigation of the electrokinetic effect.

### 4.3. DEP Characterization

Next, 0.2-, 0.5-, 1.0- and 2.0 μm particles were independently operated to evaluate the trapping effect in the proposed EDEP microfluidic chip at a given field of 90 V_pp_·cm^−1^ with the frequencies of 10–5 MHz. Table 2 shows the different microparticle sizes whether trapped or not. ‘◯’ indicates that the microparticles can be trapped, and ‘×’ indicates that they cannot be trapped. It was clear that 0.5-μm particles can be trapped with frequencies of 400–3,000 Hz, and 1.0- and 2.0-μm particles can be trapped with 100–810,000 Hz. In contrast, 0.2-μm particles cannot be trapped. Particle sizes of 0.2, 0.5, 1.0 and 2.0 μm can be easily separated by adjusting the driving frequencies.

**Figure 7.**
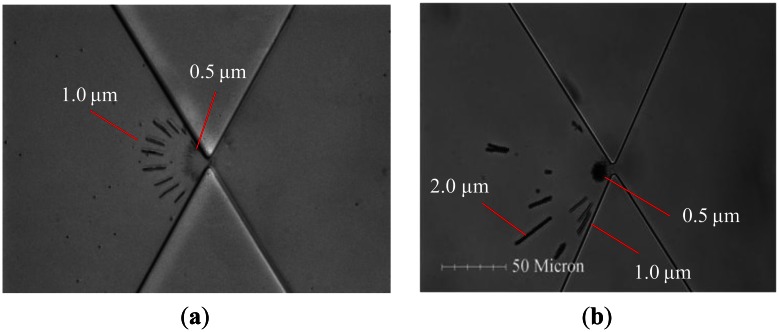
(**a**) A series of images for the trapping of 0.5- and 1.0-μm particles at the driving frequency of 400 Hz. (**b**) Separation performance of 0.5-, 1.0- and 2.0-μm particles at the driving frequency of 400 Hz. The applied potential is kept at 90 V_pp_·cm^−1^.

Finally, a solution mixed with 0.5- and 1.0 μm particles was pipetted into the reservoir, and a field of 90 V_pp_ with 400 Hz was applied to the electrodes. The microparticles continuously flowed in the presence of the EOF through the triangular insulators and were then separated in the DEP-affected area, which was close to the constricting gap, as shown in Figure 7(a). Clearly, the n-DEP force acted locally on the 0.5-μm particles approximately 10 μm from the constriction. Similarly, the DEP force acted locally on the 1.0-μm particles approximately 20∼30 μm from the constriction. Notably, the microparticles were trapped dielectrophoretically at one side of the gap, and the particle-trapped contours demonstrated the semi-circle shape in Figure 7(a). Figure 7(b) also demonstrated the separation of three different particle sizes, *i.e.*, 0.5, 1.0 and 2.0 μm, in a pair of triangular insulators. The trapped sizes of microparticles were quantitatively analyzed by ImageJ software.

## 5. Conclusions

This work has demonstrated the feasibility of utilizing electrokinesis within an EDEP microfluidic chip for microparticle separation. The separating effect depended on whether EO or DEF dominates the driving frequencies at a constant electric field of 90 V_pp_·cm^−1^. In this study, the flow produced a forward EOF when the frequencies were less than 810,000 Hz. Conversely, it produced a backward EOF when the driving frequency was beyond 810,000 Hz. Interestingly, an unstable flow, similar to a reciprocating motion, was experimentally observed at a frequency of 810,000 Hz. Experiments reveal that the optimum trapping frequencies for continuous flow are in the range of 400–3,000 Hz for 0.5 μm and 100–810,000 Hz for 1.0 and 2.0 μm particles. Furthermore, this approach can be used with continuous flow for the separation of microparticles.

## Figures and Tables

**Table 1.** Physics properties of different materials.

	***ρ* (kg·m^−3^)**	**ε _r_**	**Σ (S/m)**	**κ (W/m^2^K)**	**c (J/KgK)**
Medium	1000	80	0.07	0.613	4186
Particle	1009	2.5	0.0009	0.15	1460
PDMS	970	2.5		0.15	1460
Glass	2250	7.4		1.4	840

**Table 2.** Driving frequency *versus* different microparticle sizes.

**Hz *μ*m**	**10**	**100**	**400**	**1 k**	**3 k**	**10 k**	**100 k**	**810 k**	**1 M**	**5 M**
0.2	×	×	×	×	×	×	×	×	×	×
0.5	×	×	◯	◯	◯	×	×	×	×	×
1.0	×	◯	◯	◯	◯	◯	◯	◯	×	×
2.0	×	◯	◯	◯	◯	◯	◯	◯	×	×
